# Single measurement detection of individual cell ionic oscillations using an n-type semiconductor – electrolyte interface

**DOI:** 10.1038/s41598-018-26015-1

**Published:** 2018-05-18

**Authors:** Mariusz Pietruszka, Monika Olszewska, Lukasz Machura, Edward Rówiński

**Affiliations:** 1 Department Plant Physiology, Faculty of Biology end Environment Protection, University of Silesia, Jagiellońska 28, PL-40032 Katowice, Poland; 2 Department of Computational Physics and Electronics, Institute of Physics, University of Silesia, Katowice, Poland; 3 Institute of Materials Science, University of Silesia, Chorzów, Poland

## Abstract

Pollen tubes are used as models in studies on the type of tip-growth in plants. They are an example of polarised and rapid growth because pollen tubes are able to quickly invade the flower pistil in order to accomplish fertilisation. How different ionic fluxes are perceived, processed or generated in the pollen tube is still not satisfactorily understood. In order to measure the H^+^, K^+^, Ca^2+^ and Cl^−^ fluxes of a single pollen tube, we developed an Electrical Lab on a Photovoltaic-Chip (ELoPvC) on which the evolving cell was immersed in an electrolyte of a germination medium. Pollen from *Hyacinthus orientalis* L. was investigated *ex vivo*. We observed that the growing cell changed the (redox) potential in the medium in a periodic manner. This subtle measurement was feasible due to the effects that were taking place at the semiconductor-liquid interface. The experiment confirmed the existence of the ionic oscillations that accompany the periodic extension of pollen tubes, thereby providing – in a single run – the complete discrete frequency spectrum and phase relationships of the ion gradients and fluxes, while all of the metabolic and enzymatic functions of the cell life cycle were preserved. Furthermore, the global 1/*f*^α^ characteristic of the power spectral density, which corresponds to the membrane channel noise, was found.

## Introduction

Pollen tubes are rapidly evolving structures that are used as models in the study of plant morphogenesis, cellular differentiation, cell wall biochemistry, biomechanics and intra- and intercellular signalling^1^, which are able to respond to a combination of external or internal cues^2^. The pollen tube is a cellular protuberance that elongates *via* tip growth^2^. In order to grow, it needs to exchange mass and charge (information) with the outside world^3^ – ion homeostasis and signalling are crucial in regulating pollen tube initiation and elongation. Pollen tubes are a tailored model for studying ion dynamics at the cell biology level. Although a plethora of scientific papers has been published^3^, little is known about the timing of all of the incoming or outgoing ions that participate in pollen cell extension. Usually, a specific anion or cation transport is analysed and coupled to the growth rate alterations. Pollen tube growth is strictly dependent on these ion dynamics. Ion fluxes and cytosolic gradients of concentration have been mechanistically associated with the action of specific transporters, especially of protons^4^. Here, we propose a unique measurement that is based on energy band bending at the interface between the semiconductor and an electrolyte solution (in which the pollen tube was immersed), which is able to produce the entire spectrum of the occurrences of (periodic) ionic currents during growth.

When a semiconducting electrode is brought into contact with an electrolyte solution, a potential difference is established at the interface^5^. The variation of the electrostatic potential U(*x*) in the surface region entails a bending of the energy bands, since the potential contributes to the electronic energy with the term −eU(*x*), where e is the elementary charge. Since the elongating pollen tube exchanges charged particles (ions) with the surrounding solution, this potential difference will change at the interface accordingly. However, because the dynamics of the incoming and outgoing cation and anion fluxes are periodic in their nature, this fact should be reflected in the variation of the potential at the semiconductor-solution interface. In order to obtain reproducible experimental conditions, we developed a simple ELoPvC in which this phenomenon is utilised in order to determine the characteristic frequencies and phases of the ionic oscillations of a freely evolving *Hyacinthus orientalis* L. (hyacinth) pollen tube.

In this work a photovoltaic device was chosen to serve as a very sensitive (10^−6^ volts) detector of the dynamic electromotive force (voltage) generated by cyclic ion fluxes of an intact growing cell. In what follows, first, we prove the existence of a characteristic membrane channel noise, and then measure the ion oscillations of a hyacinth pollen tube.

## Materials and Methods

### Biological material and pollen germination

Fresh *Hyacinthus orientalis* L. pollen was obtained from plants that were grown at room temperature under stable growth conditions. To collect the pollen grains, five flowers were immersed in a 2 ml Eppendorf tube containing 0.5 ml of a pollen germination medium. The medium consisted of 10% sucrose, 10 mg/l H_3_B0_3_, 300 mg/l Ca(NO_3_)_2_, 100 mg/l KNO_3_ and 200 mg/l MgSO_4_. These substances were solved in distilled water. Average pH of the medium equalled 6.22 ± 0.005 at 23.5 ± 0.1 °C. The pollen grains were pre-incubated at 25 °C in an Eppendorf tube on a rotary device for one hour in the dark. Measurements using a CC-105 conductivity meter revealed the conductance of the medium at a level of about 0.40 μS/cm at close to 25 °C in order to ensure the proper (electrolytic) conditions during the measurements (see also Supplementary Figs 1A,B, 2 and 3A for the experimental setup).

### Measurement instruments

The auxiliary structural studies of the crystalline silicon (Si) photovoltaic cell were carried out using a Canon G11 camera, a JEOL-JSM-6480 scanning electron microscope, a PHI 5700/660 spectrometer (Physical Electronics) and an XRT 100 CCM diffractometer (EFG GmbH).

### Features of a crystalline solar cell

Supplementary Fig. 3B shows the top electrode, which consists of silver gridlines connected by a bus bar to form a double comb-shaped structure. The bottom electrode was formed by two series of silver stripes and polycrystalline aluminium (substrate, Supplementary Fig. 3C). The front surface of the Si solar cell was covered with micrometre-sized pyramid structures (textured surface, Supplementary Fig. 3D). An anti-reflection coating of zinc oxide (ZnO, thickness ~10 nm) was overlaid on the textured Si surface (Supplementary Fig. 3F). The surface roughness was determined to be ~0.2 nm. The average thickness of the Si cells was 250 ± 9 μm with an active area of 101 × 11 mm^2^. Supplementary Fig. 3G shows the backscatter Laue diffraction pattern from the crystalline plane of Miller index (100) of the Si. The Laue diffraction pattern is very symmetric with sets of diffraction spots arranged in radial shapes that revolve around the centre of the pattern (indicating a crystal Si structure).

### Voltage measurements

A single pollen with a growing pollen tube was selected under an optical microscope (Motic Microscope RED233 at a 100 × magnification). The selected pollen, which was contained in a 10 μl germination medium, was transferred onto a photovoltaic semiconductor (n-p, phosphorus 0.007% at.-boron, junction on the Si crystal, 250 μm thick, Solartec, Czech Republic) plate (Supplementary Figs 1B and 2). A droplet with a pollen was deposited onto the surface (Supplementary Fig. 1); the geometric (round) area of the liquid-chip interface was about 18 mm^2^; the contact with the electrical wires was established on the upper and lower sides (electrode) of the plate using crocodile clips. The presence of the selected pollen (Supplementary Fig. 3A) on the top of the semiconductor plate (Supplementary Fig. 3B) was checked using a stereoscopic microscope immediately after the transfer. The entire process from the transfer stabilisation of the system and the calibration of the Tektronix DMM 4040 6-1/2 Digit Precision Multimeter voltmeter after the deposition of the pollen onto the semiconducting plate took around 10 min. Data were directly collected onto an external memory drive. The entire measurement was conducted in the dark for 20 min. (because of the internal memory capacity of the instrument) at a 4.1 Hz sampling (fulfilling the Nyquist sampling criterion). Over 35 separate measurements were made using about 8 pollen tubes (one per day) with 4–5 subsequent measurements per tube. Representative outcomes are presented. The voltage measurement was possible due to the energy band bending effects at the semiconductor-electrolyte interface^5^ (see Supplementary Fig. 4, Tables 1 and 2 and Supplementary Information text passage for further details). The approximate external conditions at the time of the measurements were set to about 25 °C and 30% humidity.

### Ion oscillation measurements

**Figure 1:**
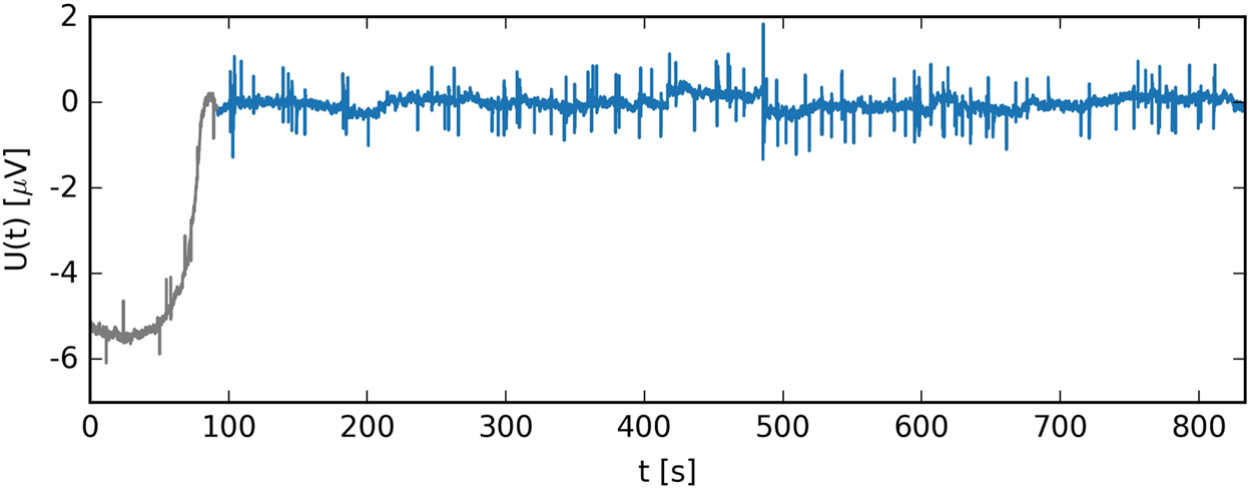
Raw time series of the electromotive force registered in the experiment. Data are visually divided into two parts. At the beginning of the experiment (solid grey line), the system has to settle down; hence, this part of the data represents transient effects. The rest of the plot (blue solid line) represents a minimally stable region of the fluctuating force *U*.

The voltage time series *U*(*t*) [μV] that was recorded during pollen tube growth revealed rather regular spikes over the duration of the experiment (Fig. 1 and Supplementary Fig. 5). In order to uncover the specific ionic fluxes that were responsible for the ion dynamics and the growth of the cell itself, a Fourier spectrum was calculated^6,7^. A control (empty, no pollen) measurement revealed no apparent oscillations of the voltage. Random voltage fluctuations around the zero base signal are unavoidable in complex biological systems. Note that strong fluctuations are the norm for a non-equilibrium steady state.

## Results

### Channel noise

More than five decades ago, Derksen and Verveen^8^ identified the power spectral density (PSD) of myelinated axons as being inversely proportional to the frequency *f*. The so-called 1/*f* (flicker or pink) noise was linked with the randomly open/close state of the membrane ion channels. This stochastic phenomenon is known as channel noise, which can be directly related to the channel conductance fluctuations^9,10^. The manifestation of flicker noise was found to reflect the complex equilibrium protein dynamics that influence channel conductance, and therefore, it is “not a fundamental property of the nonequilibrium transport phenomena”^11^. These findings were later supported using nanofabricated synthetic pores^12^. Moreover, it became clear that the 1/*f* frequency characteristics can only be linked with such a random *modus operandi*.

In the general case, the PSD of biological or synthetic membrane signals was found to follow the *f* ^α^ characteristics. The above-mentioned α = −1 reflects the motions of the channel wall constituents^9,12^, which result in the randomly open/close state of a channel. For α = −2, one can find that the channel works in a monostable open region with a constant but fluctuating ion flux^12^. On the other hand, α = 0 reflects a close or non-conducting channel and the resulting PSD is identical to that for the thermal equilibrium white noise^8^. The latter case is visible at the high-frequency end of the spectrum provided that the Nyquist frequency has been reached at the time of the measurement.

**Figure 2:**
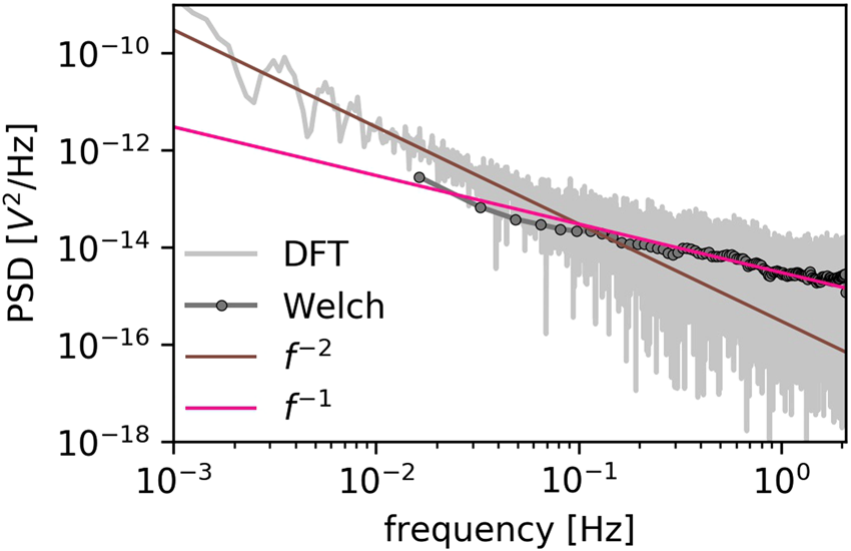
Power spectral density as a function of the frequency calculated for the complete measurement using the ELoPvC. PSD was calculated using the Welch method (grey circles) and shadowed by the results of the discrete Fourier transform (light grey in the background) manifest the 1/*f* ^2^ noise behaviour that is indicated by the solid brown line as well as the 1/*f* noise, which is designated by the straight pink line for the minimally stable region of the electromotive force.

A typical time series for an experimental setup is presented in Fig. 1. It shows an irregularly changing electromotive force as a function of time *U* = U(*t*). The signal has two clear modes – the initial growth of the force *U*, which is shown in grey, for almost one hundred seconds at the beginning and a minimally stable fluctuating signal, which is shown in blue, for the rest of the plot. Additionally, one can spot regularly appearing spikes that point upwards as well as downwards at the baseline. The results of these two regions have rather different PSD characteristics, which reflect the findings of Siwy and Fuliński^12^ (see Fig. 2 for details). The initial growth of *U*(*t*) (see the grey line in Fig. 1) corresponds with the constantly open biochannels, which results in the *f*^*−*2^ characteristics^12^. This feature is visible for the slow dynamics of the system, which are indicated by a brown solid line at the beginning of the PSD characteristics. This part of the signal matches the properties of the Brownian walk (red noise). For the main part of the data, in which the electromotive force fluctuates around the zero microvolt, the PSD that was obtained revealed a 1/*f* flicker (pink) noise, which reciprocates the randomly alternating open/close states of the channels^9,12^.

**Figure 3:**
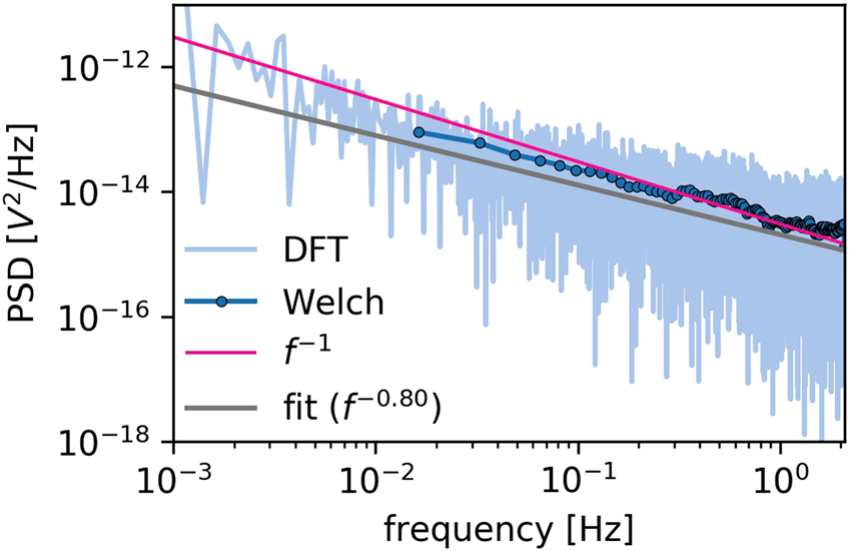
Power spectral density as a function of the frequency calculated for the currents was measured using the ELoPvC. PSD was calculated using the Welch method (blue circles) and shadowed by the results of the discrete Fourier transform (light blue in the background) manifest the 1/*f* noise behaviour for more than four decades of frequencies (feature that is highly characteristic for the membrane ion channels), which is indicated by the straight pink line for the minimally stable region of the electromotive force. The actual fit, which resulted in a slightly different power law (α = −0.791 ± 1.779 ∙ 10^−4^), is indicated by a grey line.

We now take a closer look at the main section of the signal and extract only this part of the data, which does not show any evident increase, see the blue line in Fig. 1. The PSD for this part, which was the typical measurement in our experiment, strongly supports the previous findings^9,12^, thus they present the overall 1/*f* noise characteristics at the normal operational state, cf. the pink line in Fig. 3. The actual linear fit that is presented for the selected data results in the estimated power law PSD ~ *f* ^α^, with α = −0.791 ± 1.779 ∙10^−4^ (see the grey line in Fig. 3), which is in accordance with the cited literature^8–12^.

The system under consideration is unavoidably complex. There are a number of processes that contribute to the global state of a cell and in turn to a cell’s response to external stimuli. The afore-mentioned channel dynamics are just one of many mechanisms. The actual ion fluxes through these channels naturally contribute to the overall picture. The description of the known ion oscillations is the subject of the second part of this work.

### Characteristic frequencies for ion transport

Pollens are one of the best characterised types of plant cell in many respects. However, identifying the frequencies of the ionic currents that are involved in pollen tube growth would offer increasing possibilities for the integrative comprehension of cell morphogenesis. Many reports have identified the characteristic time scales of the cations and anions that are involved separately. However, none of these reports has presented a single method that is able to produce a full frequency spectrum and phase relations for all of the participating ions. To fill this gap, we propose what follows for a growing hyacinth pollen tube based on the considerations presented above.

**Figure 4:**
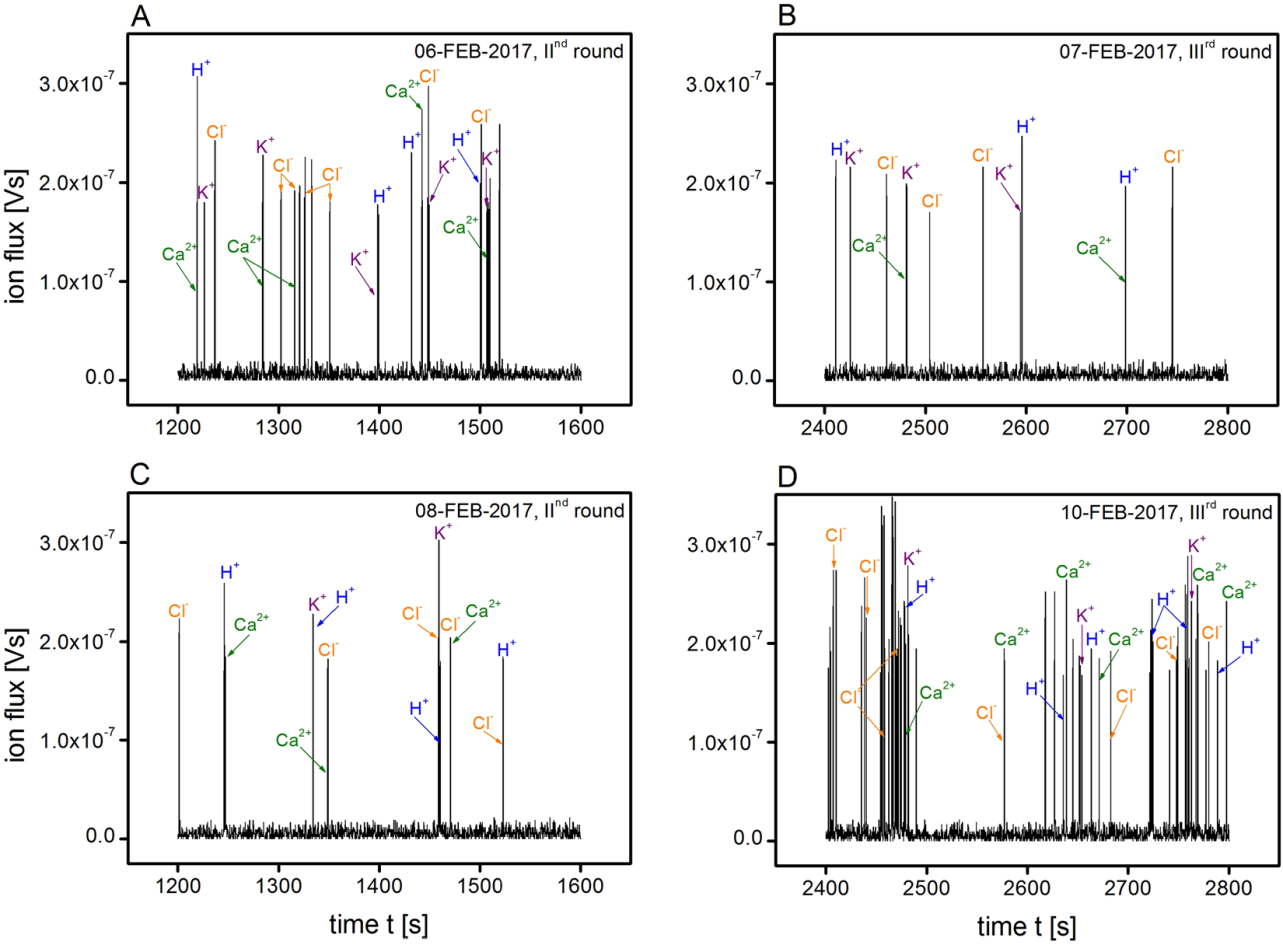
Integral spectrum as a function of duration of the growth of hyacinth pollen tube. Specific ionic fluxes (spikes) are indicated by the arrows (see also Supplementary Table 3 for further details). Since flux is an additive variable, the intensity (height) of the peaks differs.

The influx- and efflux-induced voltage oscillations of Ca^2+^ along with H^+^, K^+^ and Cl^−^ were densely sampled at about 4 Hz, which allowed the structure of the pulse to be “grasped” (in short, e.g. an average period of 15 sec, which gives about 80 full cycles in a 20-min. experiment cycle, should be easily detected in a Fourier analysis). Next, the Lorentz distribution spectrum in the time domain was calculated (Supplementary Fig. 6) and the characteristic ion-specific timings were detected (Fig. 4) in all of the conducted probes. These indicated the existence of a periodic sequence of pulses (see also Supplementary Fig. 5). The distinctive periods of the contributing ion fluxes were later compared with the literature data^13^ (Supplementary Table 3). As a result, a band of life-vital individual pulses was obtained in a single run of this rather inexpensive experiment.

**Figure 5:**
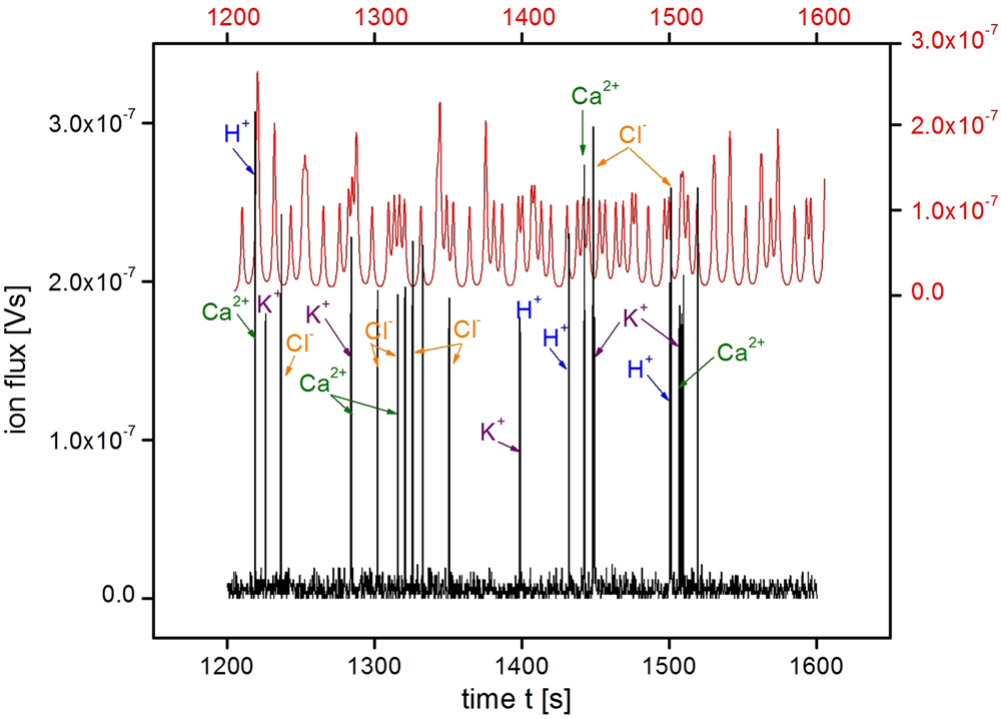
Combined spectrum of the ion fluxes that were present in the oscillations of the hyacinth pollen tube with the calculated flux pattern (red).

The result of our analysis is illustrated in the panels of Fig. 4. The phase relationships of the ion gradients and fluxes in the oscillations during hyacinth pollen tube growth, which were roughly obtained using Supplementary Fig. 6, are presented in Fig. 5, in which the sum of the signal patterns was added for comparison. The calculated time spectrum consists of several characteristic spectral lines that are indicated by arrows in Fig. 4. Each of the identified spectral lines was the peak value of a characteristic Lorenz-shaped integral spectrum (see Supplementary Information).

Since the method exclusively allowed the characteristic frequencies (periods) of the ionic currents to be discerned, it was not entirely autonomous. In order to identify all of the peaks, we had to refer to the literature. The first step towards developing an autonomous method should include the use of channel blocking substances, which is beyond the scope of our present experimental possibilities. Interestingly, the detected values were the same or very close to those that had already been identified in the above-mentioned literature as well as by Pietruszka and Haduch-Sendecka^13^. The outcomes of our method, which are presented in Figs 4 and 5, can be compared with those that were obtained by Holdaway-Clarke and Hepler^14^. The main difference is the use of a method that was similar to the one that is used to detect phase transitions using a contact electrode^15,16^. However, in the present work, the investigated sample was a droplet containing an elongating tube, while the reference electrode was a semiconducting device that sensed the changes in the pollen surroundings *via* the position of the chemical potential. It is also known that the response of an oxide surface is strong towards changes in pH – all other contributions are minor compared to this and the pH contribution could possibly mask all of the other ionic currents. Even if this were the case, the H^+^ fluctuations that are ultimately significant for the growth processes were detected using this novel method, since the resulting spatial and temporal H^+^ changes are pivotal for controlling oscillating tip growth^4,17–19^. Obviously, we do not claim that we have reached a full description of the problem as the findings come only from preliminary data. However, it does reproduce the most up-to-date published data to a great extent, and therefore, it may be correct. Needless to say, the presented method, which delivers voltage oscillations as a function of time that were generated by a growing pollen tube, is non-invasive and can be used to detect the ion fluxes of living plant cells or organs. Because of the different polarisations and signal half-widths of cations and anion-induced voltage oscillations, the measurement method using ELoPvC may become fully autonomous. However, a better voltage resolution is then indispensable.

The fact that the current measurement was performed on a single growing cell, while the latter^13^ was performed on a multicellular extending grass shoot, a coleoptile, is even more exciting. Hence, the question of whether or not the values that were obtained are accidental arises. If this (such a coincidence) is the case, similar oscillation frequencies are common in plant behaviour, thus posing a question about an interesting rule within the evolutionary context.

## Conclusion

Homeostasis is any self-regulating process by which biological systems tend to maintain stability while adjusting to conditions that are optimal for their survival (here: growth). If homeostasis is successful, growth continues. The stability that is attained is actually a dynamic equilibrium in which continuous change occurs while relatively uniform conditions prevail. Any system that is in dynamic equilibrium tends to reach a steady state. When a growing pollen is disturbed, built-in regulatory devices (e.g. ionic fluxes, protein channels) respond to these disturbances in order to establish a new balance. This feedback control is observed in the mutual dependence of incoming and outgoing ionic currents coupled with pollen growth rate oscillations^20,21^.

In this context, we can still raise the question as to how the growth rate oscillations that are evident in pollen tubes (and can be viewed with a CCD camera) correlate with those that are induced by the ionic fluxes oscillatory time series that were observed in the generated voltage. We believe that using a semiconducting voltage detector (ELoPvC), which potentially permits such an analysis, opens up new avenues for investigating the physiology of an intact growing single plant cell in perturbed^22^ (or, e.g., at different tonicity) or non-perturbed (control) environmental conditions. ELoPvC might be cautiously^23^ used to investigate single cells (such as pollens) or cells that are arranged in multicellular structures (such as root hairs, which are a second model in tip-growth studies)^24^. This would allow an ELoPvC to be used in comparative studies between different cell types to identify the universal mechanisms that are involved in the tip-growth process. Extending the knowledge about the cellular basis of the elongation of root hairs in monocots^25,26^ and dicots^27^ has indicated that the growth of the root hair tubes in *Arabidopsis thaliana* is correlated with fluctuations in the polymerisation of actin microfilaments^28^. Complementing the already known basis of tip-growth elongation with detailed measurements of ion exchange may shed new light on the regulation of tip-growth type in plants.

## Electronic supplementary material

Supplementary Information
