# Supplementary material for: Single measurement detection of individual cell ionic oscillations using an n-type semiconductor – electrolyte interface
Source: Sci Rep. 2018 May 18;8:7875. doi: 10.1038/s41598-018-26015-1 (PMC5959918; doi:10.1038/s41598-018-26015-1)
Supplement: Supplementary file 1 — Supplementary Information [file 41598_2018_26015_MOESM1_ESM.pdf]

Supplementary Information

Single measurement detection of individual cell ionic oscillations using an n-type semiconductor – electrolyte interface

Mariusz Pietruszka[1,*], Monika Olszewska[1], Lukasz Machura[2], and Edward Rówiński[3]

[1]Faculty of Biology end Environment Protection, University of Silesia in Katowice, Poland
[2]Department of Computational Physics and Electronics, Institute of Physics, University of Silesia in Katowice, Poland
[3]Institute of Materials Science, University of Silesia in Katowice, Poland
[*]mariusz.pietruszka@us.edu.pl

Measurements of time-dependent voltage registered between a semiconductor and an electrolyte containing a living pollen

Measurements of time-dependent voltage registered between a semiconductor and an electrolyte containing a living pollen It is assumed that when a biological system, like a pollen tube in the electrolyte solvent interacts with a semiconductor surface of n-type the changes in the chemical potential will give rise to corresponding modifications of the voltage[1,2]. This predictions follow from the fact that the valence (VB) and conduction (CB) bands are fixed in such a case. The phenomenon of the energy band bending depends on the energy barrier height and is a function of time. The time-dependent voltage of the contact between a semiconductor and a biological system is expressed in the form[1,3]

$$U(t) = V_p - V_{n/redox}(t) \qquad \text{(Supp. Eq. 1)}$$

$$V_{n/redox}(t) = \frac{\mu_{n/redox}(t)}{Q} \qquad \text{(Supp. Eq. 2)}$$

where $U(t)$ is the measured voltage, V_p - the p-type semiconductor (Si) potential (reference electrode), $V_{n/redox}(t)$ - the redox potential of the contact between an n-type semiconductor and a biological system, Q - the charge and $\mu_{n/redox}(t)$ - the time-dependent chemical potential. Thus, variation in the ratio of the oxidized to reduced material (redox reaction) in a biological system can cause a change in the redox energy and in turn develop an output voltage. Therefore, one essentially measures the voltage between V_p and $V_{n/redox}(t)$ potentials (Supplementary Figure 1). One can consider a particle confined to a one-dimensional lattice-periodic potential using the Schrodinger equation and Bloch theorem[4]. Thus, it is possible to formulate an approach that would allow us to derive the band bending that arises due to a contact at the interface. A band diagram of the redox/semiconductor interface is illustrated in Supplementary Tables 1 and 2 as well as in Supplementary Figure 4. It contains the results for the chemical potential calculations that was obtained using the Nernst equation.

In order to link to the literature data, where the ion fluxes were usually measured, we recall what follows. The time-dependent change in the electric flux $\phi_E(t)$ of ions is related to the time-dependent voltage $U(t')$

$$\phi_E(t) = \int_0^t U(t')dt' \qquad \text{(Supp. Eq. 3)}$$

with

$$\phi_E(t) = \int_0^t \int_A E(r,t')dSdt' \qquad \text{(Supp. Eq. 4)}$$

$$E(r) = -\nabla V(r) \qquad \Rightarrow \qquad E(r,t') = -\nabla V(r,t') \qquad \text{(Supp. Eq. 5)}$$

$$\int_A E(r,t')dS = -\int dV(r,t') = U(t') \quad \text{(Supp. Eq. 6)}$$

where A - the surface bounded by the curve Γ, t or t' denote time, $E(r,t')$ - the electric field. dS the vectorial surface element, $V(r,t')$ - the electric potential and ∇ – the nabla differential operator.

Supplementary Table 1. Characteristic parameters for the biological system and semiconductor crystal before and after contact[4–7] that was calculated using the Nernst formula. All energies are referenced to the bottom of the valence band, and the absolute electrode potential is estimated to be 4.44 ± 0.02V at 25°C for rescaling.

Type of redox reaction	Redox potentials [V]	Chemical potential [eV]		
		Redox reaction (before contact)	Silicon semiconductor (before contact)	In contact
Cl^-/Cl	1.36	5.8	5.42	5.61
Ca^{2+}/Ca	-2.9	1.54	5.42	3.48
K^+/K	-2.82	1.62	5.42	3.52

Supplementary Table 2. Specific parameters of the cell membrane and semiconductor interface together with their input values.

Type of redox reaction	Redox potentials [V]	Chemical potential [eV]		
		Redox reaction (before contact)	Silicon semiconductor (before contact)	In contact
Cl^-	-65	4.435	5.42	4.927
Ca^{2+}	29	4.529	5.42	4.974
K^+	-90	4.410	5.42	4.915

Supplementary Table 3. Periods cross-reference table. References in square brackets as in Pietruszka and Haduch-Sendecka, 2015[8,9] (see main text). The lowest frequencies from the literature data were taken into account.

Ion flux	Period T [s]	Frequency f = 1/T [Hz]	References
K^+	56 ± 0.5	0.017 ± 0.001	[22] Messerli et al., 1999[10] - Fig. 5A
Ca^{2+}	32 ± 0.5	0.031 ± 0.001	[22] Messerli et al., 1999[10] - Fig. 7C<br>[23] Messerli et al., 2000[11] - Fig. 8B<br>[29] Liu et al., 2010[12] - Fig. 3A
H^+	31 ± 0.5	0.032 ± 0.001	[5] Holdaway-Clark and Hepler, 2003[13] - Fig. 3<br>[22] Messerli et al., 19999 - Fig. 4A<br>[29] Liu et al., 201011 - Fig. 3B
H^+	11 ± 0.5	0.091 ± 0.001	[8] Zonia et al., 2002[14] - Fig. 1B

References

1. Bard, A. J., Bocarsly, A. B., Fan, F. R. F., Walton, E. G. & Wrighton, M. S. The concept of fermi level pinning at semiconductor/liquid junctions. consequences for energy conversion efficiency and selection of useful solution redox couples in solar devices. *J. Am. Chem. Soc.* **102**, 3671–3677 (1980).

2. Sjulstok, E., Olsen, J. M. H. & Solov'yov, I. A. Quantifying electron transfer reactions in biological systems: what interactions play the major role? *Sci. reports* **5**, 18446 (2015).
3. Nakayama, Y. Universal time-dependent deformations of schrödinger geometry. *J. High Energy Phys.* **2010**, 102 (2010).
4. Holgate, S. A. *Understanding solid state physics* (cRc Press, 2009).
5. Atkins, P. W. *Physical Chemistry* (W.H. Freeman and Company, New York, 1997).
6. Lide, D. R. *CRC handbook of chemistry and physics* (CRC Press, 2006).
7. Vanysek, P. Electrochemical series. *Handb. Chem. Phys.* **92** (2011).
8. Pietruszka, M. & Haduch-Sendecka, A. Ion frequency landscape in growing plants. *PloS one* **10**, e0138839 (2015).
9. *See also Supplementary Information Table 1 therein*. Erratum: The authors take this opportunity to correct the previously published data - please note the erroneous value for period T = 6.7 ± 3.0s, which should correctly read for the frequency $f = 0.149$Hz.
10. Messerli, M. A., Danuser, G. & Robinson, K. R. Pulsatile influxes of h+, k+ and ca2+ lag growth pulses of lilium longiflorum pollen tubes. *J. Cell Sci.* **112**, 1497–1509 (1999).
11. Messerli, M. A., Créton, R., Jaffe, L. F. & Robinson, K. R. Periodic increases in elongation rate precede increases in cytosolic ca2+ during pollen tube growth. *Dev. biology* **222**, 84–98 (2000).
12. Liu, J., Piette, B. M., Deeks, M. J., Franklin-Tong, V. E. & Hussey, P. J. A compartmental model analysis of integrative and self-regulatory ion dynamics in pollen tube growth. *PLoS One* **5**, e13157 (2010).
13. Holdaway-Clarke, T. L. & Hepler, P. K. Control of pollen tube growth: role of ion gradients and fluxes. *New Phytol.* **159**, 539–563 (2003).
14. Zonia, L., Cordeiro, S., Tupỳ, J. & Feijó, J. A. Oscillatory chloride efflux at the pollen tube apex has a role in growth and cell volume regulation and is targeted by inositol 3, 4, 5, 6-tetrakisphosphate. *The Plant Cell* **14**, 2233–2249 (2002).

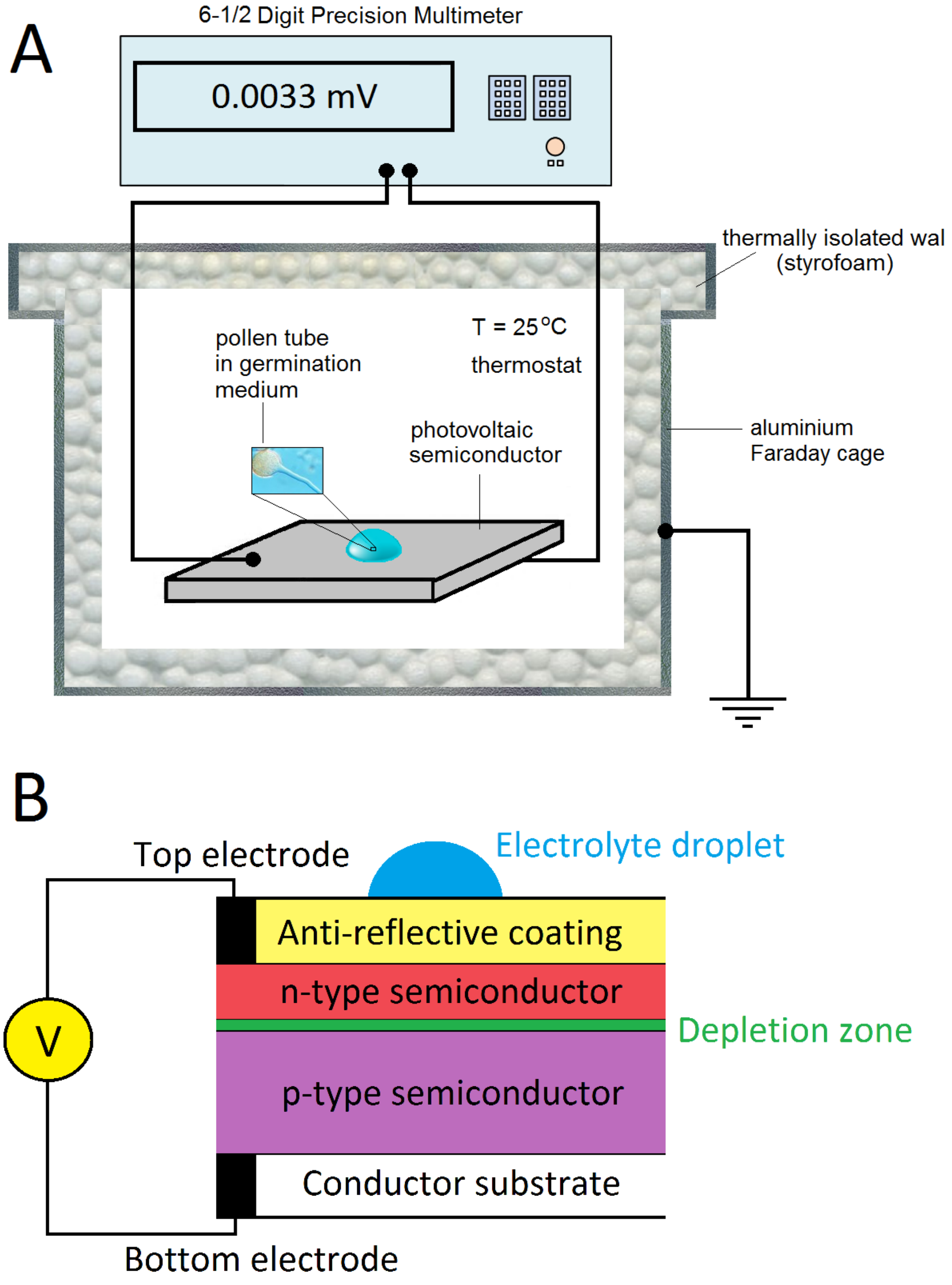

Supplementary Figure 1. (A) The experimental scheme. The pollen tube is growing in a (electrolytic) germination medium on a semiconductor plate. The EMF is measured by a voltmeter. The Faraday cage is grounded (B) Device structure of the solar cell/electrolyte droplet interface (ELoPvC), see main text.

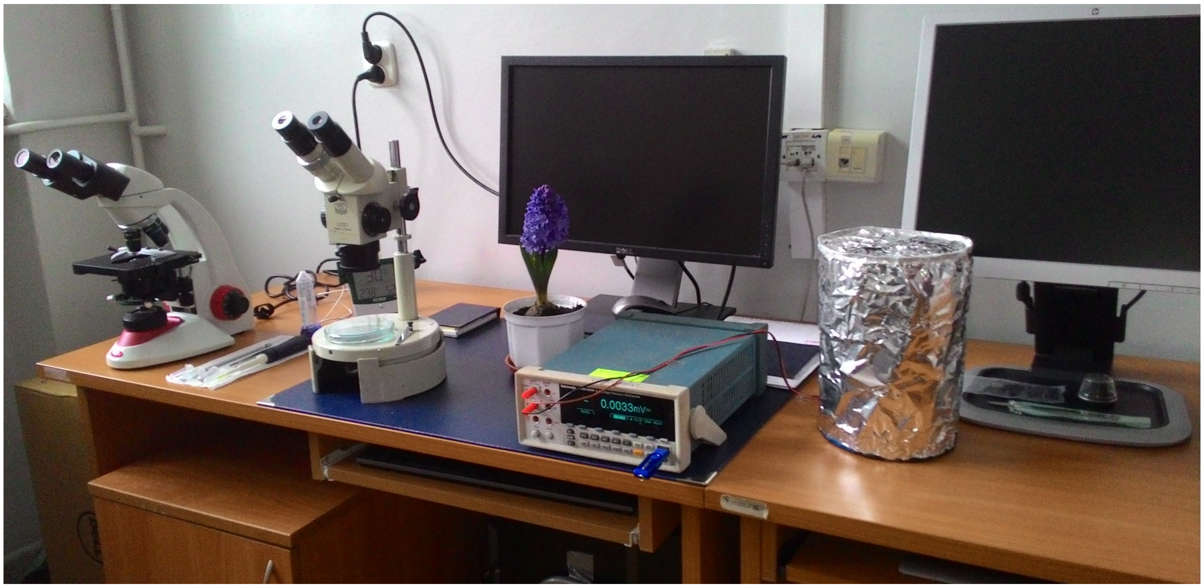

Supplementary Figure 2. The experimental setup.

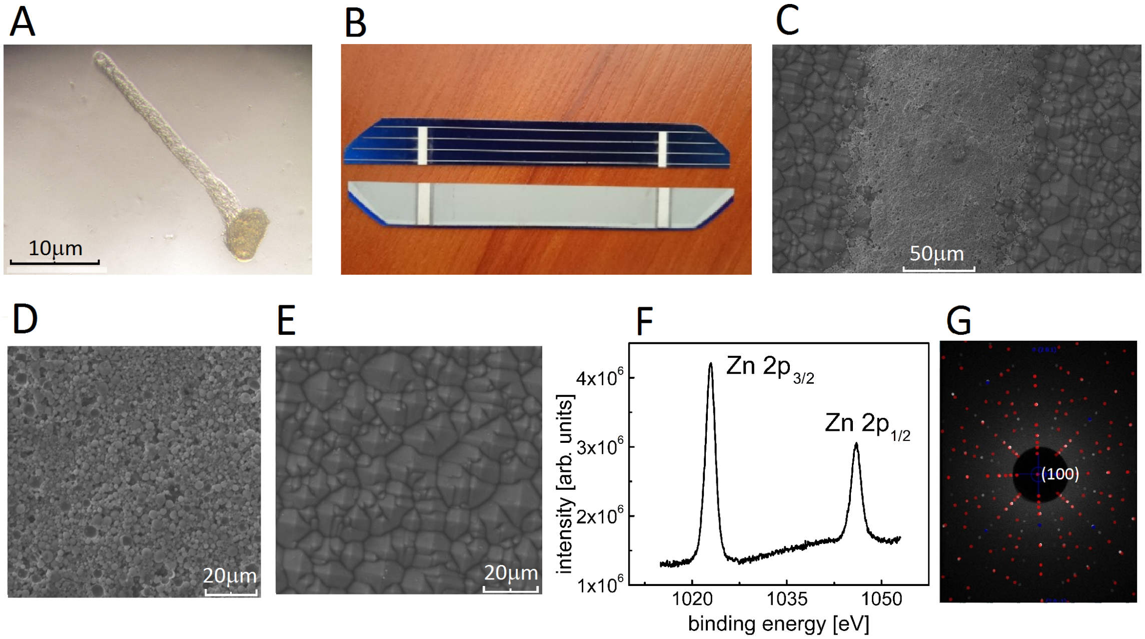

Supplementary Figure 3. (A) The hyacinth pollen growing in a germination medium (B) Photographs of top and bottom silicon solar cell (C) Micrograph of a silver electrode (D) Micrograph of aluminium bottom substrate (E) Micrograph of n-type semiconductor surface of textured silicon layer covered by anti-reflective ZnO thin layer (F) X-ray photoelectron core lines of Zn $2p_{3/2}$ and Zn $2p_{1/2}$ of ZnO layer (G) Backscatter Laue diffraction pattern from a Si (100) crystal.

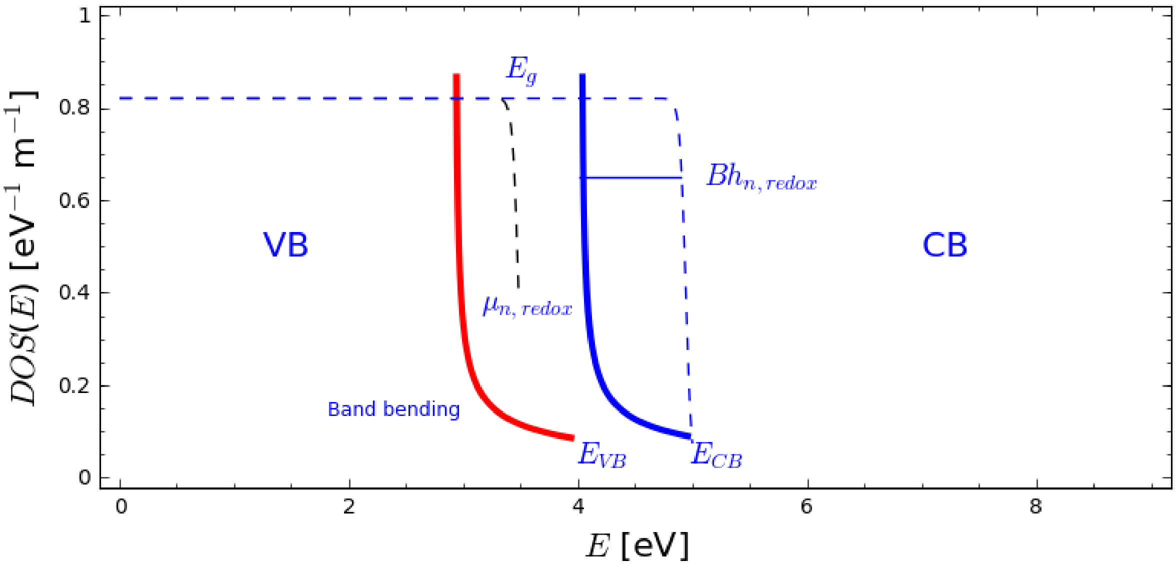

Supplementary Figure 4. Band diagram for the contact between the n-type silicon semiconductor surface and the biological redox system of Ca^{2+} /Ca ($\mu_{n/redox}$ energy level equals 3.48 eV, see also SI Table 2 for the remaining species). DOS(E) denotes the density of the states and E_{VB} is the maximum energy of the valence band, E_{CB} is the characteristic energy of the conduction band and Bh denotes the energy barrier height. E_g stands for the energy gap.

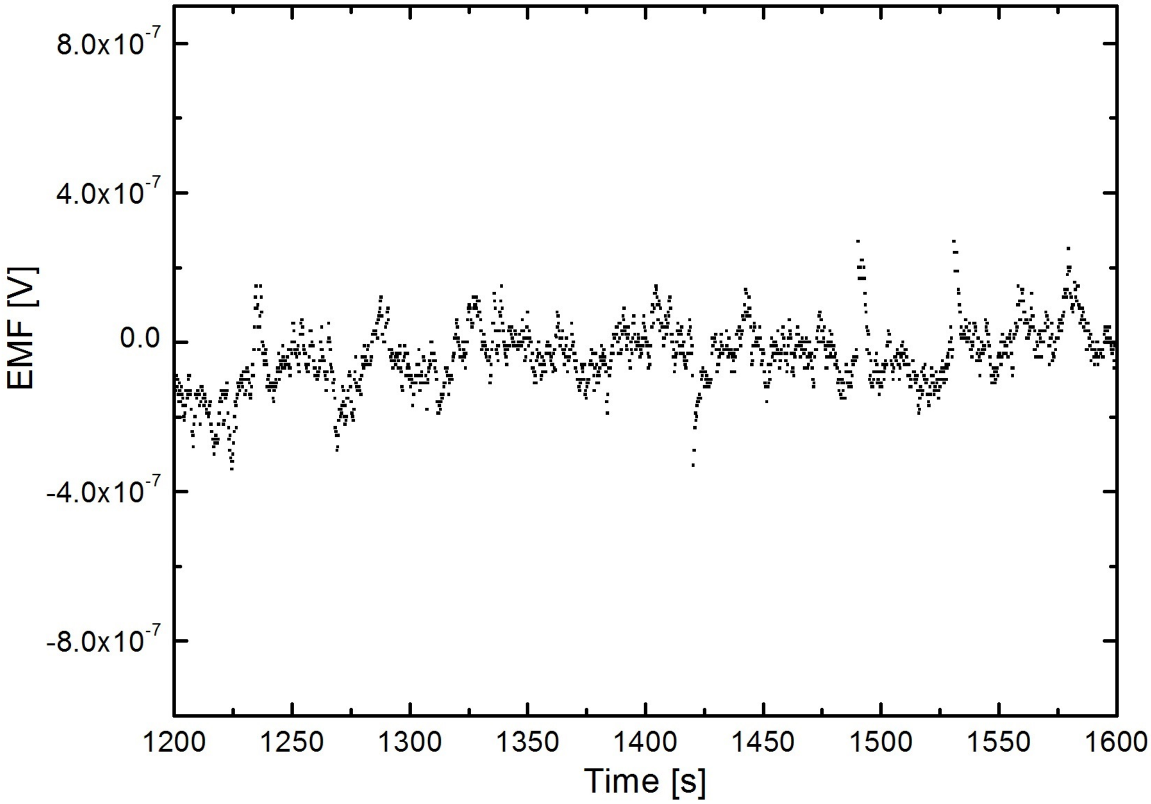

Supplementary Figure 5. Voltage (EMF) oscillations as a function of time as generated by a growing pollen tube - the raw experimental data. The electrolyte solution with a pollen deposited onto a photovoltaic plate.

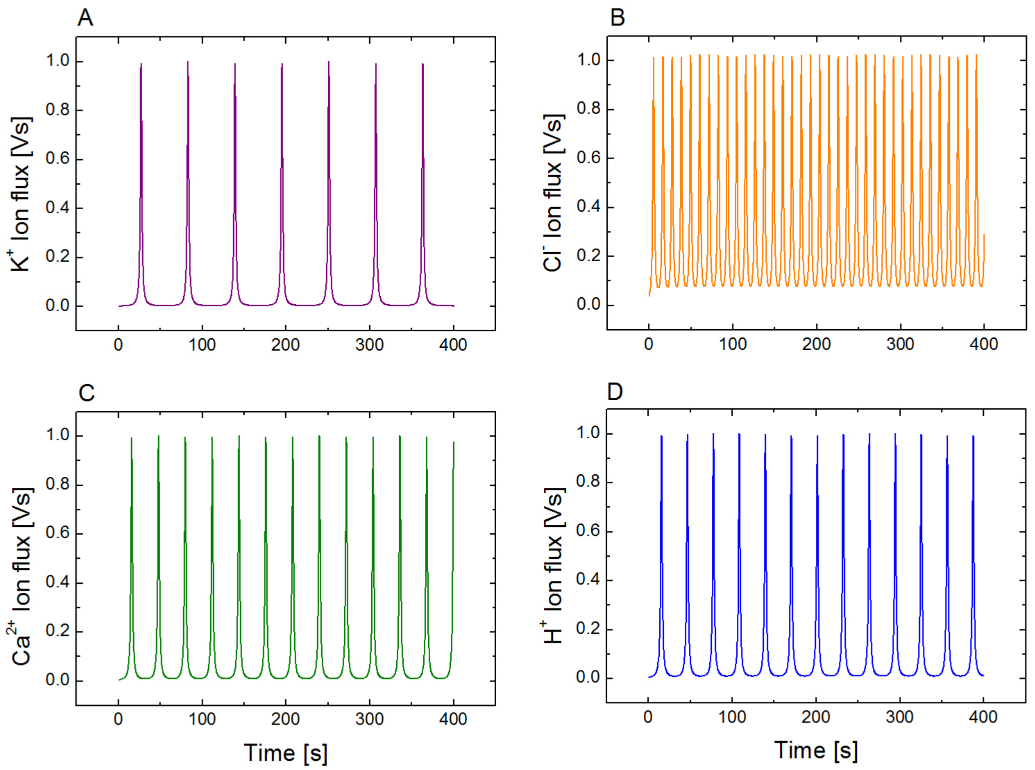

Supplementary Figure 6. The patterns of ion fluxes, calculated from supplementary Eq. 2, used to identify data from experiments. Patterns calculated with the help of the Lorentz function $\Phi_{ion}(t) = 1/[(t - T_{ion})^2 + w^2]$, where $T_{ion} = (n + ^1/_2)t$, $n = 0, 1, 2, 3 \ldots$, $w = 1$ and ion = K^+ , Cl^-, Ca^{2+} and H^+, respectively.
